# Comparative Analysis of Laparoscopic Sleeve Gastrectomy with and Without Prior Endoscopic Intragastric Balloon Insertion: Examining Stomach Volumetry, Histopathologic Changes, Hormonal Levels, and Postoperative Outcomes

**DOI:** 10.1007/s11695-025-07907-4

**Published:** 2025-05-13

**Authors:** Mohamed Hany, Frits Berends, Eman Sheta, Anwar Ashraf Abouelnasr, Ann Samy Shafiq Agayby, Ahmed Zidan, Bart Torensma, Edo Aarts

**Affiliations:** 1 Department of Surgery, Medical Research Institute, Alexandria University, Alexandria, Egypt; 2 Madina Women’s Hospital, Department of Surgery, Alexandria, Egypt; 3 Department of Surgery, WeightWorks Clinics, Amersfoort, Netherlands; 4 Department of Clinical Epidemiology, Erasmus MC, Rotterdam, Netherlands

**Keywords:** Intragastric balloon, Laparoscopic sleeve gastrectomy, Stomach volumetry, Postoperative outcomes, Leptin, Ghrelin, Food tolerance

## Abstract

**Background:**

The effects of prior intragastric balloon (IGB) placement on stomach volumetry, surgical technique, and outcomes in laparoscopic sleeve gastrectomy (LSG) patients are unclear.

**Methods:**

This prospective cohort study analyzed stomach histology, gastric volume, and hormonal markers in 90 LSG patients (45 with prior IGB, 45 without). We assessed stomach wall thickness, fibrosis, smooth muscle density, and ghrelin-positive cells, along with intraoperative parameters like stapler cartridge use and operative time. Postoperative outcomes, including weight loss and food tolerance (FT), were compared between groups at 6 months and 1 year.

**Results:**

In the 6th month and 1st year, the two groups had no differences in weight, BMI, and %TWL before and after the Inverse Propensity Score-Weighted adjustment. The IGB group had significantly increased muscular thickness, smooth muscle cell count, and fibrosis (*p* < 0.001) but similar mucosa thickness and inflammation. Preoperative stomach and resected specimen volumes were higher in the IGB group (*p* < 0.001). Both groups showed slight increases by 1 year, with no significant FT differences. Furthermore, no significant difference in postoperative complications was noted. Hormonal changes were observed, including lower leptin levels in the IGB group throughout.

**Conclusion:**

While prior intragastric balloon (IGB) placement induces significant volumetry changes and hormone levels, it does not affect surgical outcomes—including postoperative complications, weight loss, resolution of associated medical problems, the duration of IGB placement, or the interval between IGB removal and LSG surgery—compared to those without IGB.

**Supplementary Information:**

The online version contains supplementary material available at 10.1007/s11695-025-07907-4.

## Introduction

Obesity is a growing global issue with serious health risks. Various weight loss methods include lifestyle changes, medical treatments, surgery, and endoscopic alternatives [1]. Many patients with obesity do not qualify for surgery but need to lose weight for health benefits. Even among eligible patients, few choose surgery due to perceived risks. This has led to a demand for less invasive weight loss treatments such as intragastric balloons (IGBs). IGBs can aid those ineligibles for surgery, offer non-surgical options, or prepare patients with severe obesity for surgery. IGB treatment typically lasts 6 months for most systems and is most effective within a comprehensive weight loss program [2, 3]. Despite 35 years of use, IGBs have not become a widely used treatment due to moderate weight loss, while side effects are common. These side effects include abdominal discomfort and sometimes even gastric ulceration and intestinal obstruction [4–6].

Understanding of structural changes in the stomach resulting from IGB implantation remains limited. It is reasonable to hypothesize that retaining a foreign object within a muscular organ for 6 months may alter the stomach wall structure. However, few studies have specifically examined these potential changes following IGB therapy [7, 8]. Data on stomach structural changes due to IGBs are limited [9, 10]. This study analyzes the histological structure of stomach samples from LSG patients, comparing those with and without prior IGB therapy. It also seeks to test the correlations between preoperative parameters, intraoperative data, and 1-year postoperative outcomes in both groups.

## Materials and Methods

### Study Design

This prospective cohort study was conducted in two centers and included patients who underwent LSG between December 2022 and June 2023. The study was approved by the ethics committee and performed in accordance with the ethical standards of the 1964 Declaration of Helsinki. All patients provided informed consent. Ethical board registration is IORG0008812 (E/C.S/N.R15.2022).

### Study Objectives

Patients were divided into two groups: those who had previously undergone endoscopic intragastric balloon placement (IGB group) and those without (non-IGB group). The IGB placement served as a standalone weight loss intervention rather than a bridge to surgery. All patients in the IGB group had previously undergone balloon therapy at another clinic as a non-surgical approach to weight management.

### Endpoint

This study aimed to analyze the histological structure of surgically removed stomach samples, explicitly focusing on the corpus in patients who underwent LSG.

### Inclusion Criteria

The study included participants who were 18 years old or above and fulfilled the eligibility criteria based on the American Society for Metabolic and Bariatric Surgery (ASMBS) guidelines [1].

### Exclusion Criteria

Exclusion criteria are patients with a *Helicobacter pylori* infection, peptic ulcer disease, smokers, previous MBS, patients who underwent swallowable (4-months) balloon insertion, those who dropped out during the follow-up, or those who refused to comply with the study’s protocol.

### Data Collection

Data were collected preoperatively, 6 months, and 1 year after the operation.

Data consisted of demographic and operative data and blood assessments of ghrelin and leptin levels. Stomach volumetry and upper endoscopy were performed. Length of hospital stay, readmission, and complications according to the Clavien-Dindo (CD) classification were recorded [11].

### Weight Loss Measurements

%TWL was calculated using the following formula: %TWL = (initial body weight − current body weight)/initial weight × 100%. The ideal body weight (IBW) was calculated based on an ideal BMI of 25 kg/m^2^ using the following formula: IBW = 25 × (height in m)^2^ [12, 13]. %EWL was calculated using the following formula: %EWL = (initial body weight − current body weight)/(initial body weight − IBW) × 100%.

#### Food Tolerance

Food tolerance (FT) assessment was performed using the one-page questionnaire to assess the overall patient’s tolerance to food, with scores ranging from 1 to 27 [14].

### Hormonal Measurements

Peripheral blood samples were collected before surgery and 6 and 12 months postoperatively to measure the fasting levels of the hormones (ghrelin and leptin). Serum samples were allowed to clot at 18–22 °C for 30 min and then centrifuged at 4000 rpm for 10 min at 4 °C. Hormones were determined using ELISA (EIA-2935) (DRG International, Inc. Springfield NJ, USA).

### Surgical Technique

A sleeve gastrectomy was initiated 4–5 cm proximal to the pylorus using two 60-mm black cartridges with an Ethicon Echelon EndoFlex stapler, followed by gold and blue cartridges over a 40-Fr calibration tube extending to the angle of His. The staple line was reinforced with 3/0 PDS absorbable V-loc sutures at the sleeve’s upper part and 2/0 non-absorbable PBT V-loc sutures (Covidien, Mansfield, MA, USA) at the corpus. The resected stomach was removed through the left flank trocar site without drainage (full description Appendix 1).

### Radiology Assessments

To assess volumetry changes after IGB placement, expressed in volume (mL^3^), that could influence gastric sleeve construction and anatomical outcomes, a multislice spiral computed tomography (MSCT) was applied. All patients underwent MSCT scans pre-surgery and at 6 and 12 months post-surgery. Imaging was conducted on a 64-channel MSCT helical GE Emotion machine (General Electric, WI, USA), with a low-dose scan of 0.12 cm slice width completed in approximately 10 s (Appendix 2) [15, 16].

### Volume Assessment Resected Stomach

For the resected stomach, the gastric volume was measured with a small opening created at the antral end of the excised stomach. Tap water was slowly injected via a Toomey syringe until the stomach reached maximum capacity. The water was then transferred to a graded container for precise volume measurement.

### Histological and Immunohistochemical Analysis

**Fig. 1:**
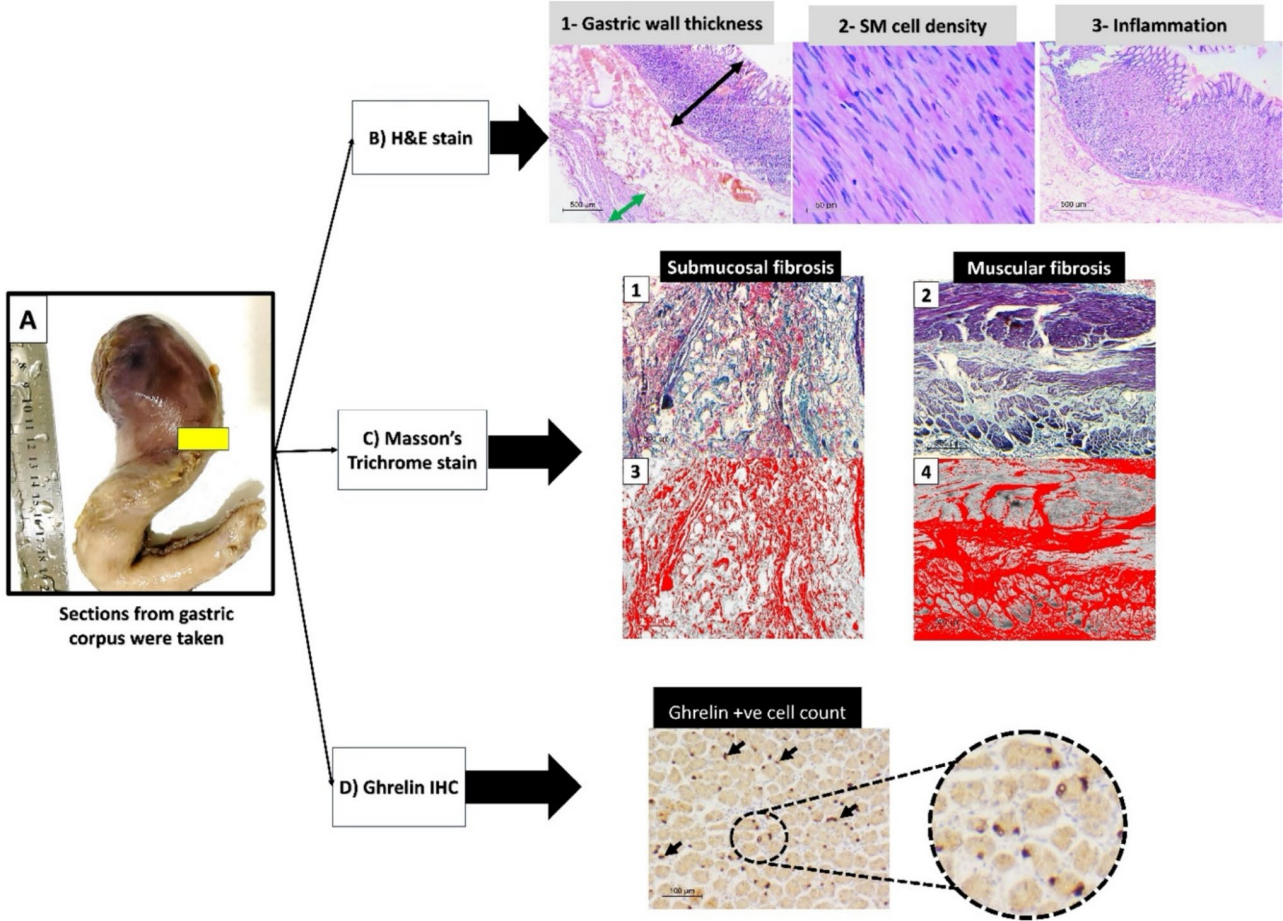
Assessed tissue parameters in gastrectomy specimens: (A) sections (yellow area) from gastric corpus were taken from gastrectomy specimens for processing into paraffin blocks. (B) H&E-stained sections were done to (1) measure gastric mucosal (black line) and musculosa propria (green line) thickness in microns at × 40 power. (2) Count of smooth muscle (SM) nuclei in diagonally cut smooth muscle in × 400 power to calculate SM density. (3) Assessment of gastric wall inflammation within mucosa. (C) Masson trichrome stained section to assess degree of fibrosis. Collagen bundles are stained blue in both (1) submucosa and (2) musculosa propria. Using image analysis software, photos were edited (3 and 4) and area of fibrosis (highlighted in red) was calculated precisely as percentage of total area examined. (D) Immunostaining (IHC) by anti-ghrelin antibody was done. Photos of mucosa at × 200 power were taken to count positive cells (black arrows). Higher magnification (× 400) highlights brown stained nucleated endocrine cells in the mucosa

Various staining techniques assessed the stomach wall thickness, smooth muscle density, fibrosis, and ghrelin-positive cell counts. H&E staining was performed on formalin-fixed gastric segments to evaluate mucosa and muscularis propria thickness, with smooth muscle density quantified via high-power field imaging (full description in Appendix 3) (Fig. 1A–D) [17–19].

### Statistical Analysis

Descriptive and inferential statistics were employed. Categorical variables were expressed as frequencies and percentages *n* (%). Continuous variables were represented as mean and standard error (SE). An Inverse Propensity Score-Weighted (IPSW) analysis was conducted to balance baseline patient characteristics using the Weighting and Analysis of Nonequivalent Groups (TWANG) package. A gradient boosting machine (GBM) model using an average treatment effect on the treated (ATT) design was used to weigh patients from the non-IGB group based on their similarity to those in the IGB group. The number of GBM trees was optimized using the plot of the average absolute standardized mean difference (SMD) of all covariates vs a number of iterations. We found that a minimum average a SMD for balancing was reached with 2950 trees.

Pearson’s chi-square test or Fisher’s exact test was used, as appropriate, to compare categorical variables among different groups. Independent samples *t*-tests were used to compare continuous variables between groups. Generalized Estimated Equations (GEE) were utilized to compare the IGB and non-IGB groups at different follow-up points. The effects of IGB duration and time between removal and LSG using the stomach tissue parameters were examined separately using univariate linear regression (unadjusted) and then in multiple linear regression analyses where each outcome was regressed on IGB duration and time between IGB removal to LSG simultaneously. Statistical significance was set at *p* ≤ 0.05. All analyses were performed using R software, version 4.2.2.

### Sample Size

A power analysis was conducted using the “pwr” package in R to determine the required sample size for the study [20]. Given the limitations of prior studies and to detect meaningful differences in outcomes [7, 8], we based our analysis on a large effect size (*d*) of 0.8. The power analysis was structured for a two-sample *t*-test with a two-sided alternative hypothesis, a desired power of 0.8, and a significance level (*α*) of 0.05, yielding a sample size of 26 participants per group. To account for potential lower effect sizes and anticipated loss to follow-up, we conservatively included 45 patients per group.

## Results

### Baseline Demographics

This study analyzed 90 patients: 45 with IGB and 45 without IGB. The complete flowchart with lost to follow-up is in Appendix 4.

The mean ± SE age was 38.6 ± 1.3 years, and 59 (65.6%) were female. The pre-LSG average body mass index (BMI) was mean ± SE 44.3 ± 0.7 kg/m^2^ for the IGB group and 44.1 ± 0.7 kg/m^2^ for the non-IGB group. No significant differences were observed between the IGB and non-IGB groups across all baseline demographics. The mean ± SE duration of IGB placement was 5.7 ± 0.1 months. The mean ± SE time between IGB removal and LSG was 3.3 ± 1.6 months (range 2–8), and the mean recurrence weight gain after nadir was 25.8 ± 13.0 kg (Table 1).

**Table 1:** Inverse probability score weighting

Variable	Unadjusted				Adjusted by IPSW			
	IGB (*n* = 45)	Non-IGB (*n* = 45)	aSMD	*p*	IGB (*n* = 45)	Non-IGB (*n* = 14)	aSMD	*p*
Age, mean ± SE	37.8 ± 1.3	39.4 ± 1.3	0.178	0.394	37.8 ± 1.3	37.3 ± 1.9	0.062	0.809
Sex, *n* (%)								
Female	31 (68.9%)	28 (62.2%)	0.142	0.657	31 (68.9%)	10 (71.4%)	0.02	0.932
Male	14 (31.1%)	17 (37.8%)			14 (31.1%)	4 (28.6%)		
Anthropometrics								
Height, mean ± SE	1.7 ± 0.0	1.7 ± 0.0	0.047	0.833	1.7 ± 0.0	1.6 ± 0.0	0.148	0.531
Weight, mean ± SE	122.1 ± 3.1	120.5 ± 2.5	0.076	0.690	122.1 ± 3.1	120.1 ± 4.3	0.094	0.711
BMI, mean ± SE	44.3 ± 0.7	44.1 ± 0.7	0.041	0.840	44.3 ± 0.7	44.4 ± 1.2	0.016	0.957
Associated medical problems, *n* (%)								
One or more associated medical problems	13 (28.9%)	18 (40.0%)	0.242	0.375	13 (28.9%)	5 (35.7%)	0.067	0.779
DM	9 (20.0%)	8 (17.8%)	0.055	1.000	9 (20.0%)	2 (14.3%)	0.153	0.504
Hypertension	8 (17.8%)	6 (13.3%)	0.115	0.771	8 (17.8%)	2 (14.3%)	0.08	0.724
OSAS	1 (2.2%)	0 (0.0%)	0.149	1.000	1 (2.2%)	0 (0.0%)	0.149	1.000
Hypothyroidism	1 (2.2%)	2 (4.4%)	0.149	1.000	1 (2.2%)	0 (0.0%)	0.001	1.000
Cardiac ischemia	0 (0.0%)	2 (4.4%)	0.299	0.494	0 (0.0%)	0 (0.0%)	0.153	1.000
Pre-surgical endoscopy, *n* (%)								
Pre endo hiatal hernia (2–4 cm)	4 (8.9%)	5 (11.1%)	0.077	1.000	4 (8.9%)	2 (14.3%)	0.052	0.634
GERD A	3 (6.7%)	2 (4.4%)	0.088	1.000	3 (6.7%)	1 (7.1%)	0.07	1.000
Gastritis	4 (8.9%)	1 (2.2%)	0.232	0.361	4 (8.9%)	1 (7.1%)	0.164	1.000

### Resected Stomach Volume, Specimen Volume, and Tissue Parameters

**Fig. 2:**
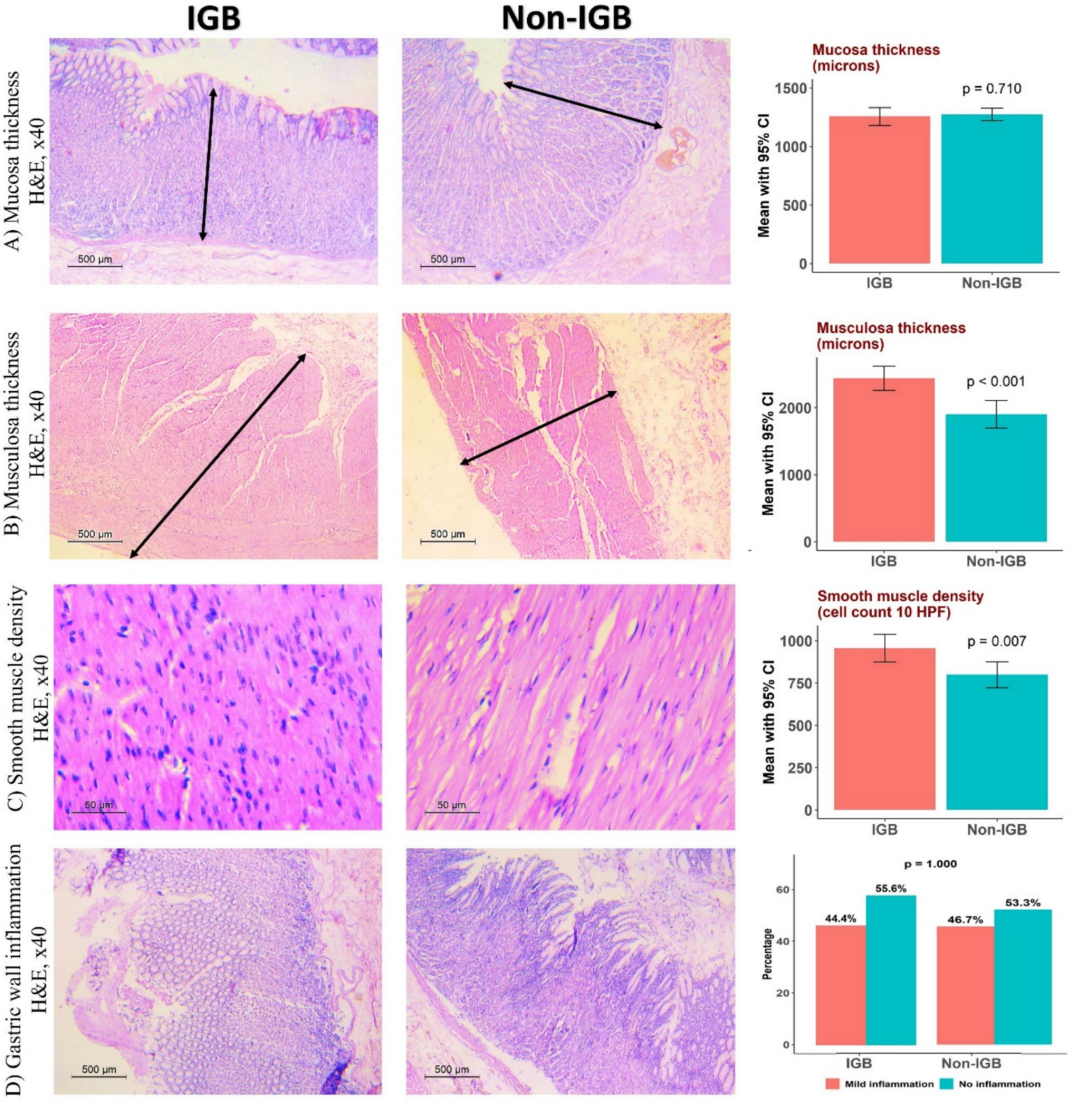
Tissue parameters assessed in hematoxylin and eosin (H&E) stained sections of gastric body in both IGB and non-IGB patients. **A** No statistically significant difference was seen in mucosal thickness between both groups (black arrows). **B** Thicker musculosa propria was seen in IGB group (black arrows). Also noted, **C** increased smooth muscle density in the same group seen as increase smooth muscle nuclei density in × 400 fields. **D** Mild or no inflammation was seen in both groups. IGB, intragastric balloon; HPF, high-power field

The IGB group had greater musculosa thickness (*p* < 0.001 in both analyses), smooth muscle cell density (unadjusted: *p* = 0.007, adjusted: *p* < 0.001), and fibrosis percentage in smooth muscle and submucosa (*p* < 0.001 in both analyses). However, mucosa thickness, ghrelin cell density, and inflammation did not differ significantly (Table 2, Fig. 2).

**Table 2:** Operative and resected tissue parameters data before and after inverse probability score weighting

Variable	Unadjusted			Adjusted by IPSW		
	IGB (*n* = 45), mean ± SE	Non-IGB (*n* = 45), mean ± SE	*p*	IGB (*n* = 45), mean ± SE	Non-IGB (*n* = 14), mean ± SE	*p*
Operative time (minutes)	60.5 ± 1.2	57.4 ± 0.9	0.045*	60.5 ± 1.2	57.3 ± 1.0	0.044*
Number of reloads	6.3 ± 0.1	5.3 ± 0.1	< 0.001*	6.3 ± 0.1	5.3 ± 0.1	< 0.001*
LOH days	2.2 ± 0.1	2.1 ± 0.1	0.830	2.2 ± 0.1	2.3 ± 0.1	0.438
Concomitant hiatal hernia repair, *n* (%)	4 (8.9%)	5 (11.1%)	1.000	4 (8.9%)	2 (14.3%)	0.634
Difference in operative time (IGB-non-IGB) adjusted for concomitant hiatal hernia repair (95% CI)	3.1 (0.1, 6.1)		0.039*	3.3 (0.2, 6.3)		0.039*
Stomach histopathological parameters and specimen volume						
Specimen volume (mL)	929.9 ± 7.9	703.2 ± 8.5	< 0.001*	929.9 ± 7.9	701.5 ± 12.1	< 0.001*
Tissue parameters						
Mucosa thickness (microns)	1256.7 ± 38.5	1274.3 ± 27.1	0.710	1256.7 ± 38.5	1247.1 ± 35.6	0.854
Musculosa thickness (microns)	2440.2 ± 91.5	1902.4 ± 105.2	< 0.001*	2440.2 ± 91.5	1821.3 ± 126.8	< 0.001*
Smooth muscle density cell count (10 HPF)	955.3 ± 41.5	799.6 ± 38.9	0.007*	955.3 ± 41.5	766.5 ± 31.1	< 0.001*
Smooth muscle fibrosis percent	47.5 ± 1.1	25.0 ± 0.9	< 0.001*	47.5 ± 1.1	25.0 ± 1.2	< 0.001*
Submucosal fibrosis percent	41.5 ± 0.9	27.7 ± 1.0	< 0.001*	41.5 ± 0.9	26.8 ± 1.2	< 0.001*
Ghrelin cells density cell count (× 200 PF)	23.9 ± 2.1	20.9 ± 1.5	0.256	23.9 ± 2.1	20.0 ± 1.5	0.139
Inflammation						
No inflammation	25 (55.6%)	24 (53.3%)	1.000	25 (55.6%)	7 (50%)	0.133
Mild inflammation	20 (44.4%)	21 (46.7%)		20 (44.4%)	7 (50%)	

*Significant differences (*p*= <0.05)

### Operative Data

Operative time was only 3.2 min longer in the IGB group compared to the non-IGB group before and after IPSW adjustment (60.5 ± 1.2 vs 57.3 ± 1.0, *p* = 0.044 after IPSW adjustment). Length of hospital stay (LOH) and concomitant hiatal hernia repair prevalence showed no significant differences between groups before and after adjustment (Table 2).

### Weight, BMI, %EWL, and %TWL Over Time

In the 6 th month and 1 st year, there were no differences in weight, BMI, and %TWL between the two groups before and after IPSW adjustment. Only %EWL was slightly higher in the non-IGB group at 6 months (MD = − 1.9 (95% CI − 3.0 to − 0.7), *p* = 0.001), but not significant at 1 year (MD = − 1.9 (95% CI − 4.0, 0.1), *p* 0.064) (Table 3).

**Table 3:** Comparisons of weight, BMI, %EWL, and %TWL overtime between IGB and non-IGB group before and after inverse probability score weighting (estimated by GEE)

Variable	Unadjusted				Adjusted by IPSW			
	IGB (*n* = 45), mean ± SE	Non-IGB (*n* = 45), mean ± SE	MD (95% CI)	*p*	IGB (*n* = 45), mean ± SE	Non-IGB (*n* = 14), mean ± SE	MD (95% CI)	*p*
Weight								
6 th month	101.3 ± 2.2	99.3 ± 1.8	2.0 (− 45.6, 49.6)	0.934	101.3 ± 2.2	98.8 ± 2.8	2.6 (− 48.8, 53.9)	0.921
1 st year	85.7 ± 1.7	84.0 ± 1.6	1.7 (− 119.8, 123.3)	0.978	85.7 ± 1.7	83.3 ± 2.2	2.4 (− 62.7, 67.5)	0.943
BMI								
6 th month	36.8 ± 0.5	36.3 ± 0.4	0.5 (− 3.6, 4.5)	0.827	36.8 ± 0.5	36.5 ± 0.7	0.3 (− 2.3, 2.9)	0.820
1 st year	31.1 ± 0.3	30.7 ± 0.2	0.5 (− 3.2, 4.1)	0.800	31.1 ± 0.3	30.7 ± 0.4	0.4 (− 2.1, 2.9)	0.754
%EWL								
6 th month	38.9 ± 0.5	40.8 ± 0.3	− 1.9 (− 3.0, − 0.7)	0.001*	38.9 ± 0.5	40.9 ± 0.4	− 2.0 (− 3.3, − 0.8)	0.002*
1 st year	68.5 ± 0.7	70.3 ± 0.7	− 1.8 (− 3.7, 0.0)	0.052	68.5 ± 0.7	70.4 ± 0.8	− 1.9 (− 4.0, 0.1)	0.064
%TWL								
6 th month	16.7 ± 0.4	17.4 ± 0.3	− 0.7 (− 1.8, 0.3)	0.168	16.7 ± 0.4	17.5 ± 0.6	− 0.8 (− 2.2, 0.6)	0.254
1 st year	29.3 ± 0.7	30.1 ± 0.6	− 0.8 (− 2.6, 1.0)	0.369	29.3 ± 0.7	30.2 ± 1.1	− 0.9 (− 3.4, 1.6)	0.463

*Significant differences (*p*= <0.05)

### Ghrelin Level, Leptin Level, Stomach Volume, and Food Tolerance

**Fig. 3:**
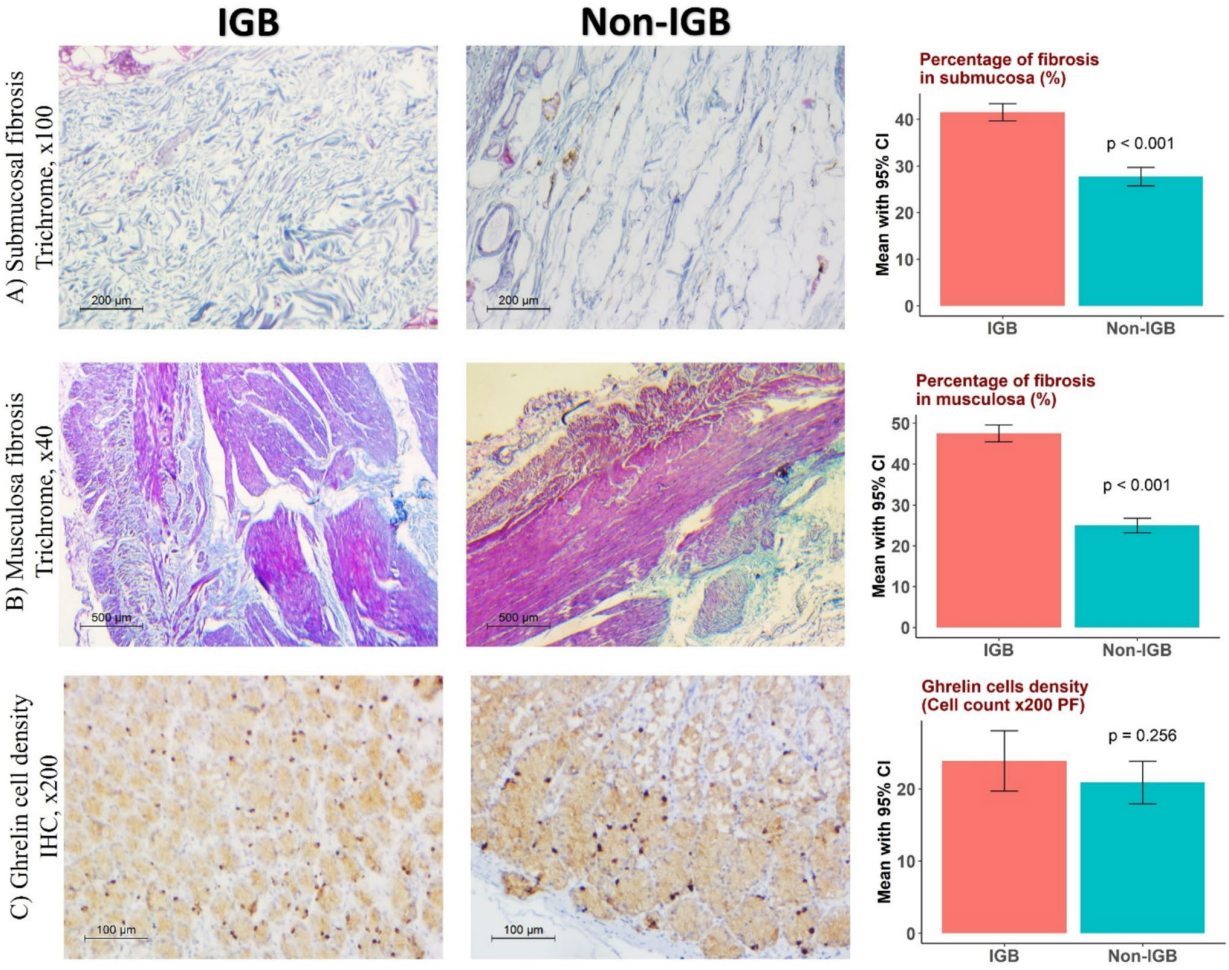
**A**, **B** Tissue parameters assessed in Masson trichrome stained sections of gastric body in both IGB and non-IGB patients. Collagen bundles are stained blue while smooth muscles are stained red. Increased fibrosis is seen in both submucosa and musculosa propria in IGB group in comparison to non-IGB. **C** Ghrelin immune stained sections show scattered dark brown positive neuroendocrine cells in crypts base. Ghrelin cell density is slightly higher in IGB group however statistically insignificant. IGB, intragastric balloon; PF, power field

Ghrelin levels were significantly higher (after IPSW) in the IGB group, with a mean of 343.2 ± 10.1 pg/mL, compared to the non-IGB group, 313.9 ± 10.5 pg/mL. No statistically significant differences were observed between the two groups before and after IPSW adjustments after surgery at 6 months and 1 year (Table 4, Fig. 3).

**Table 4:** Comparisons of ghrelin level, leptin level, stomach volume, and food tolerance overtime between IGB and non-IGB group before and after inverse probability score weighting (estimated by GEE)

Variable	Unadjusted				Adjusted by IPSW			
	IGB (*n* = 45), mean ± SE	Non-IGB (*n* = 45), mean ± SE	MD (95% CI)	*p*	IGB (*n* = 45), mean ± SE	Non-IGB (*n* = 14), mean ± SE	MD (95% CI)	*p*
**Ghrelin (pg/mL)**								
Before surgery	343.2 ± 10.1	317.8 ± 9.1	25.3 (− 1.0, 51.7)	0.059	343.2 ± 10.1	313.9 ± 10.5	29.2 (0.9, 57.6)	0.043*
1 st month	215.2 ± 7.1	214.7 ± 6.9	0.5 (− 18.7, 19.6)	0.960	215.2 ± 7.1	219.5 ± 8.0	− 4.3 (− 25.1, 16.6)	0.686
6 th month	229.2 ± 6.7	229.1 ± 8.3	0.1 (− 20.6, 20.8)	0.990	229.2 ± 6.7	224.7 ± 10.3	4.5 (− 19.3, 28.3)	0.712
1 st year	238.1 ± 6.4	238.0 ± 9.2	0.0 (− 21.7, 21.8)	0.997	238.1 ± 6.4	233.6 ± 11.3	4.5 (− 20.8, 29.7)	0.727
**Leptin (ng/mL)**								
Before surgery	84.5 ± 0.4	95.3 ± 0.4	− 10.8 (− 11.9, − 9.6)	< 0.001*	84.5 ± 0.4	95.2 ± 0.5	− 10.6 (− 11.9, − 9,4)	< 0.001*
6 th month	44.7 ± 0.5	54.7 ± 0.4	− 10.0 (− 11.2, − 8.8)	< 0.001*	44.7 ± 0.5	54.6 ± 0.5	− 9.9 (− 11.1, − 8.6)	< 0.001*
1 st year	24.6 ± 0.5	35.4 ± 0.4	− 10.9 (− 12.1, − 9.7)	< 0.001*	24.6 ± 0.5	35.4 ± 0.5	− 10.8 (− 12.1, − 9.5)	< 0.001*
**Stomach volume (mL**^**3**^**)**								
Before surgery	1108.9 ± 7.9	863.9 ± 6.7	245.0 (224.9, 265.1)	< 0.001*	1108.9 ± 7.9	866.8 ± 7.4	242.1 (221.1, 263.1)	< 0.001*
6 th month	135.6 ± 0.5	146.6 ± 0.4	− 11.0 (− 12.2, − 9.8)	< 0.001*	135.6 ± 0.5	146.5 ± 0.4	− 10.9 (− 12.2, − 9.6)	< 0.001*
1 st year	154.5 ± 0.4	166.0 ± 0.7	− 11.6 (− 13.2, − 9.9)	< 0.001*	154.5 ± 0.4	166.0 ± 0.9	− 11.6 (− 13.6, − 9.6)	< 0.001*
**Food tolerance**								
6 th month	23.1 ± 0.1	21.5 ± 0.3	1.5 (1.0, 2.1)	< 0.001*	23.1 ± 0.1	21.6 ± 0.4	1.5 (0.7, 2.3)	< 0.001*
1 st year	23.9 ± 0.3	23.9 ± 0.2	− 0.1 (− 0.8, 0.6)	0.850	23.9 ± 0.3	23.9 ± 0.4	0.0 (− 0.9, 0.8)	0.934

*Significant differences (*p*= <0.05)

The groups had a statistically significant difference in leptin levels before and after IPSW adjustments before surgery in the IGB vs non-IGB group mean ± SE 84.5 ± 0.4 vs 95.2 ± 0.5, *p* ≤ 0.001.

**Fig. 4:**
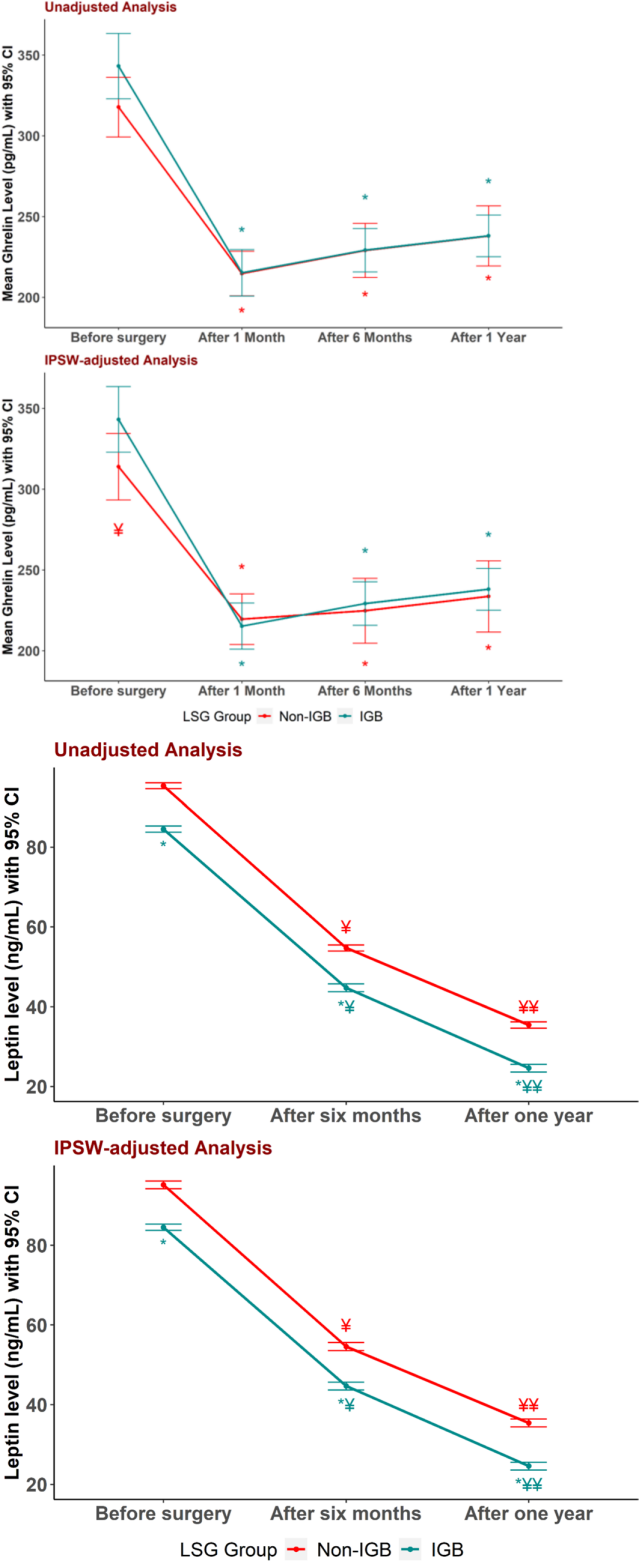
Ghrelin and Leptin level over time with and without IPSW adjustments. Data are presented as mean (95% CI). ^¥^*p* < 0.001 vs before surgery. ^¥¥^*p* < 0.001 1 year vs 6 months. *p < 0.05 IGB vs non-IGB

At 6 months and 1 year post-surgery, both groups exhibited significant reductions in leptin levels (all *p* < 0.001). Leptin levels decreased significantly from 6 months to 1 year in both groups (all *p* < 0.001). Throughout this period, the IGB group consistently had significantly lower leptin levels than the non-IGB group in both unadjusted and adjusted analyses (Table 4, Fig. 4).

The IGB group’s stomach volume was significantly higher than the non-IGB group, adjustment by IPSW (MD 242.1 mL, 95% CI 221.1 to 263.1, *p* < 0.001). At 6 months postoperative, the IGB group had a significantly smaller stomach volume compared to the non-IGB group, with a mean difference (MD) of − 11.0 mL (95% CI − 12.2 to − 9.8, *p* < 0.001) before and − 10.9 mL (95% CI − 12.2 to − 9.6, *p* < 0.001) after IPSW adjustment. At 1 year, both groups showed a slight increase in stomach volume (*p* < 0.001), yet the IGB group remained lower by 11.6 mL (95% CI 9.9 to 13.2, *p* < 0.001) unadjusted, and 11.6 mL (95% CI 9.6 to 13.6, *p* < 0.001) adjusted (Table 4).

At 6 months, the FT score was significantly higher in the IGB group compared to the non-IGB group, both before (MD = 1.5, 95% CI 1.0 to 2.1, *p* < 0.001) and after IPSW adjustment (MD = 1.5, 95% CI 0.7 to 2.3, *p* < 0.001). After 1 year, no significant differences in FT were observed between the groups (Table 4).

#### Early Complications (≤ 30 days)

Eight (17.8%) cases of early complications were reported in the IGB group and seven (15.65%) cases in the non-IGB group (*p* > 0.05).

Seven cases of early complications were reported in the IGB group, compared to six cases in the non-IGB group. These complications included superficial wound infection, vomiting, and respiratory infection. All affected patients received outpatient medications, classifying these complications as Clavien-Dindo (CD) class I (26.6%).

#### Late Complications (> 30 days)

Late complications were not different between groups and included the diagnosis of a hiatal hernia during a screening endoscopy 1 year after LSG in 13 (28.9%) patients from the IGB group and 12 (26.7%) patients from the non-IGP group (*p* = 1.000) (Table 5).

**Table 5:** Early and late postoperative complications, Dindo classes, and readmission rates between IGB and non-IGB group before and after inverse probability score weighting

Variable	Unadjusted			Adjusted by IPSW		
	IGB (*n* = 45)	Non-IGB (*n* = 45)	*p*	IGB (*n* = 45)	Non-IGB (*n* = 14)	*p*
Early complications (≤ 30 days)						
Gastrointestinal bleeding (melena)	1 (2.2%)	1 (2.2%)	1.000	1 (2.2%)	0 (0.0%)	1.000
DVT	1 (2.2%)	0 (0.0%)	1.000	1 (7.1%)	0 (0.0%)	0.237
Respiratory infection	0 (0.0%)	1 (2.2%)	1.000	0 (0.0%)	1 (7.1%)	0.237
Vomiting	1 (2.2%)	3 (6.7%)	0.616	1 (2.2%)	2 (14.3%)	0.151
Wound infection	5 (11.1%)	2 (4.4%)	0.434	3 (21.4%)	2 (4.4%)	0.081
Late complications (> 30 days)						
Denovo GERD	5 (11.1%)	4 (8.9%)	1.000	5 (11.1%)	1 (7.1%)	1.000
Hiatal hernia	13 (28.9%)	12 (26.7%)	1.000	3 (6.7%)	0 (0.0%)	1.000
Port site hernia	1 (2.2%)	0 (0.0%)	1.000	1 (2.2%)	0 (0.0%)	1.000
Dindo classification for early complications						
Class I	6 (13.3%)	6 (13.3%)	1.000	5 (11.1%)	1 (7.1%)	1.000
Class II	2 (4.4%)	1 (2.2%)	1.000	1 (2.2%)	1 (7.1%)	0.421
Endoscopy at year 1						
Denovo GERD (A, B) LA classification	5 (11.1%)	4 (8.9%)	1.000	5 (11.1%)	1 (7.1%)	1.000
Hiatal hernia	13 (28.9%)	12 (26.7%)	1.000	3 (6.7%)	0 (0.0%)	1.000
Readmission	2 (4.4%)	1 (2.2%)	1.000	1 (2.2%)	0 (0.0%)	0.149

### Resolution/Improvement in Associated Medical Problems

Before and after surgery, there were no significant differences in the prevalence of associated medical problems between the two groups. Following LSG, both groups showed significant resolution of diabetes mellitus (DM): from 20 to 4.4% in the IGB group (*p* = 0.023) and from 17.8 to 4.4% in the non-IGB group (*p* = 0.041), with no significant difference between the groups (Table 6).

**Table 6:** Comparisons of associated medical problems between IGB and non-IGB group before and after LSG before and after inverse probability score weighting

Associated medical problems	Time	Unadjusted			Adjusted by IPSW		
		IGB (*n* = 45)	Non-IGB (*n* = 45)	*p*^b^	IGB (*n* = 45)	Non-IGB (*n* = 28)	*p*^b^
DM	Before	9 (20.0%)	8 (17.8%)	1.000	9 (20.0%)	2 (14.3%)	0.504
	After	2 (4.4%)	2 (4.4%)	1.000	2 (4.4%)	1 (7.1%)	0.564
	*p*^a^	0.023*	0.041*		0.023*	0.317	
Hypertension	Before	8 (17.8%)	6 (13.3%)	0.771	8 (17.8%)	2 (14.3%)	0.724
	After	3 (6.7%)	2 (4.4%)	1.000	3 (6.7%)	0 (0.0%)	1.000
	*p*^a^	0.074	0.134		0.074	0.157	
OSAS	Before	1 (2.2%)	0 (0.0%)	1.000	1 (2.2%)	0 (0.0%)	1.000
	After	0 (0.0%)	0 (0.0%)	1.000	0 (0.0%)	0 (0.0%)	1.000
	*p*^a^	1.000	NA		1.000	NA	
Hypothyroidism	Before	1 (2.2%)	2 (4.4%)	1.000	1 (2.2%)	0 (0.0%)	1.000
	After	0 (0.0%)	0 (0.0%)	1.000	0 (0.0%)	0 (0.0%)	1.000
	*p*^a^	1.000	0.480		1.000	NA	
Cardiac ischemia	Before	0 (0.0%)	2 (4.4%)	0.494	0 (0.0%)	0 (0.0%)	1.000
	After	0 (0.0%)	1 (2.2%)	1.000	0 (0.0%)	0 (0.0%)	1.000
	*p*^a^	NA	1.000		NA	NA	

*Significant differences (*p*= <0.05)

*p*^a^: within the group

*p*^b^: between the groups

### Regression Analyses Investigating the Impact of Intragastric Balloon on Gastric Wall Histologic Parameters

The univariate and multiple linear regression analyses examined the impact of IGB on various stomach tissue outcomes, including musculosa thickness, smooth muscle density, smooth muscle fibrosis, submucosal fibrosis, ghrelin cell density, and pre-surgical ghrelin levels. Results indicated that neither the duration of IGB placement nor the interval between IGB removal and LSG surgery was associated with the stomach tissue outcomes before surgery (Table 7).

**Table 7:** Results of the univariate (unadjusted) and multiple (adjusted) linear regression analyses for the effect of IGB duration and time between IGB removal and LSG on the stomach tissue parameters

Outcome	Predictor	Unadjusted		Adjusted	
		*β* (95% CI)	*p*	*β* (95% CI)	*p*
Mucosa thickness microns	IGB duration (months)	− 34.4 (− 124.1, 55.3)	0.443	− 9.4 (− 104.8, 86.0)	0.843
	Time between IGB removal and LSG (months)	38.3 (− 8.8, 85.4)	0.108	36.5 (− 14.8, 87.8)	0.159
Musculosa thickness microns	IGB duration (months)	− 189.7 (− 396.4, 17.1)	0.071	− 161.2 (− 385.1, 62.8)	0.154
	Time between IGB removal and LSG (months)	73.4 (− 39.8, 186.7)	0.198	41.5 (− 78.9, 161.9)	0.491
Smooth muscle density cell count 10 HPF	IGB duration (months)	− 38.8 (− 135.5, 57.8)	0.422	− 18.4 (− 122.2, 85.5)	0.723
	Time between IGB removal and LSG (months)	33.5 (− 17.9, 84.8)	0.196	29.8 (− 26.0, 85.6)	0.287
Smooth muscle fibrosis percent	IGB duration (months)	− 0.5 (− 3.0, 1.9)	0.666	− 0.4 (− 3.1, 2.3)	0.759
	Time between IGB removal and LSG (months)	0.3 (− 1.1, 1.6)	0.694	0.2 (− 1.3, 1.6)	0.802
Submucosal fibrosis percent	IGB duration (months)	0.9 (− 1.3, 3.1)	0.394	0.9 (− 1.5, 3.3)	0.474
	Time between IGB removal and LSG (months)	− 0.3 (− 1.5, 0.9)	0.624	− 0.1 (− 1.4, 1.2)	0.850
Ghrelin cells density cell count × 200 PF	IGB duration (months)	− 1.8 (− 6.8, 3.2)	0.469	− 3.3 (− 8.6, 1.9)	0.205
	Time between IGB removal and LSG (months)	− 1.6 (− 4.2, 1.1)	0.231	− 2.3 (− 5.1, 0.6)	0.114
Ghrelin pre 1 week before surgery	IGB duration (months)	− 10.0 (− 33.4, 13.4)	0.393	− 14.3 (− 39.5, 10.9)	0.259
	Time between IGB removal and LSG (months)	− 3.4 (− 16.1, 9.2)	0.588	− 6.3 (− 19.8, 7.3)	0.357

## Discussion 

This prospective cohort study was conducted between patients who had previously undergone endoscopic intragastric balloon placement (IGB group) in another clinic and those who had not (non-IGB group).

### Changes in Stomach Wall vs Time Between Surgery After IGB Placement

The IGB group showed a notable increase in muscular thickness, smooth muscle cell count, and fibrosis in both the smooth muscle and submucosa (*p* < 0.001). However, mucosa thickness and inflammation were similar across groups.

A study by Rzepa et al. examined histological changes in 16 patients with prior IGB insertion as a bridge surgery procedure compared to the control group (14 patients) who had one-step LSG. They reported significantly greater stomach wall thickness, increased submucosal fibrosis, and higher inflammation in the IGB group compared to controls. These changes were attributed to a shorter interval (1–3 months) between balloon removal and surgery to prevent weight recurrence, likely insufficient for complete structural reversal to prevent recurrence [8, 21]. Patrzyk et al. examined stomach histology in 12 patients post-IGB implantation and subsequent LSG, compared to only four control patients without IGB implantation. The balloons were in place for an average of 29 weeks, with an 8-week interval between balloon removal and LSG. They found no effect on stomach wall thickness from prior IGB implantation but observed changes in tissue composition, including hypertrophy and fibrosis, potentially reducing tissue elasticity and compressibility [18]. The differences between our study and previous findings may stem from varied intervals between IGB removal and subsequent surgery and differences in exclusion criteria, such as *Helicobacter pylori* infection or smoking status. Therefore, further well-matched powered studies are necessary to counteract and test all confounding factors.

### Weight Loss

Long-term outcomes of IGB therapy vary widely. While some patients benefit from repeated endoscopic treatments, others transition to medical therapy or MBS. In one study, patients initially lost an average of 12.6 kg with IGB, but there was a recurrence of weight gain of 6.5 kg over 2 years [22]. Similarly, most patients do not sustain IGB-related weight loss long-term, aligning with other time-limited weight loss methods [23, 24]. In our study, the mean EWL was 34.3 ± 2.5%, and the TWL was 11.0 ± 0.6% after IGB placement. At the 6 th month and 1 st year follow-up, the two groups had minimal weight, BMI, and %TWL differences. However, there was a significant difference in %EWL at the 6 th month, with the non-IGB group achieving a higher percentage of EWL (38.9 ± 0.5 vs 40.8 ± 0.3, *p* 0.001).

One possible mechanism for this could be desensitization to the adipokine leptin. Therefore, the lower leptin levels after IGB preconditioning could contribute to attenuated weight loss after MBS. Throughout our study, the IGB group consistently exhibited significantly lower leptin levels than the non-IGB group [25].

### Hormone Levels

IGB placement alters stomach wall distensibility and volume, and studies indicate that weight loss from IGB may correlate with temporary hormonal shifts, including reduced leptin and increased ghrelin [26]. However, these hormonal changes do not consistently predict weight loss sustainability. Our study showed no difference in GPC expression between IGB and non-IGB groups, reinforcing findings that LSG effects are not directly linked to ghrelin levels. Preoperatively, ghrelin levels were higher in the IGB group (343.2 ± 10.1 pg/mL vs 313.9 ± 10.5 pg/mL in non-IGB). Still, postoperatively, ghrelin levels declined similarly in both groups, with no impact on weight recurrence after 1 year, suggesting alternative mechanisms contribute to long-term weight maintenance.

Leptin levels were lower pre-surgery in the IGB group, with both groups showing significant reductions post-surgery. These results confirm leptin sensitization may occur due to prior IGB-induced weight loss [27]. While Pellitero et al. found no difference in leptin between weight recurrence (WR) and non-WR groups [28], Santo et al. observed higher baseline leptin in patients with WR after RYGB and LSG [29]. Sustained weight loss likely results from reduced stomach capacity, improved dietary habits, increased physical activity, and personal motivation to maintain weight for well-being or to avoid regain rather than relying solely on hormonal factors.

### Preoperative Stomach Volume, Food Tolerance, and Resected Sleeve Volume

In our study, preoperative stomach volume and resected specimen volume were significantly higher in the IGB group compared to the non-IGB group (*p* < 0.001). Six months post-surgery, the IGB group’s stomach volume was smaller than the non-IGB group’s. In one study, the authors discovered a significant positive association between gastric pouch volume and various factors such as weight loss (*p* value = 0.04), BMI reduction (*p* value < 0.0001), and ∆weight (*p* = 0.013) at 1 year [30]. Following LSG, the larger resected volume and slightly dilated remaining stomach in IGB patients may result in a “bounce-back” effect, where the remaining stomach volume adjusts and reduces postoperatively [31]. Although a 12 mL difference post-surgery was statistically significant, it was minor—approximately the size of a tablespoon—and unlikely to have clinical relevance. However, factors such as antrum size and bougie size substantially impacted long-term outcomes by influencing the remaining stomach’s capacity and distensibility. This suggests that while we could measure minor differences, they did not translate into meaningful effects on weight loss or other clinical outcomes. Instead, factors like hormonal changes, physical activity, dietary behaviors, and gastric emptying may impact weight loss more than resected stomach volume alone [32].

### Limitations

This study has several limitations. First, IGB insertion and removal were performed outside our center, limiting our knowledge about the type of IGB used and relying on patient-reported data. Second, there was inconsistency in the timing of IGB insertion, removal, and subsequent surgery, which could impact outcomes. Third, the follow-up period was limited to 1 year, and longer-term outcomes remain unknown. Additionally, we did not assess inflammatory markers in gastric tissues, although inflammation appeared mild. Our study exclusively included patients with endoscopically inserted IGBs, excluding those with swallowable balloons. While no patients in this cohort used swallowable balloons, they would have been excluded due to their different insertion methods. Endoscopic IGB insertion allows for direct visualization of the stomach wall, assessment for pathology, and biopsy, if necessary, advantages not afforded by swallowable balloons. This approach helps prevent undetected pre-existing tissue changes or pathologies that could affect outcomes.

## Conclusion

While prior intragastric balloon (IGB) placement induces significant volumetry changes and hormone levels, it does not affect surgical outcomes—including postoperative complications, weight loss, resolution of associated medical problems, the duration of IGB placement, or the interval between IGB removal and LSG surgery—compared to those without IGB. The appropriate use of IGB must be further investigated for patients who are eligible for MBS.

## Supplementary Information

Below is the link to the electronic supplementary material.

Supplementary file1 (DOCX 14 KB)

Supplementary file2 (DOCX 13 KB)

Supplementary file3 (DOCX 14 KB)

Supplementary file4 (DOCX 182 KB)

## Data Availability

Data with the corresponding author available.
