# Supplementary material for: Comparative Analysis of Laparoscopic Sleeve Gastrectomy with and Without Prior Endoscopic Intragastric Balloon Insertion: Examining Stomach Volumetry, Histopathologic Changes, Hormonal Levels, and Postoperative Outcomes
Source: Obes Surg. 2025 May 13;35(6):2039–52. doi: 10.1007/s11695-025-07907-4 (PMC12129852; doi:10.1007/s11695-025-07907-4)
Supplement: Supplementary file 1 — Supplementary file1 (DOCX 14 KB) [file 11695_2025_7907_MOESM1_ESM.docx]

**Surgical technique**

In patients who had previously undergone IGB insertion, each sleeve gastrectomy procedure was started 4-5 cm proximal to the pylorus, utilizing continuous applications of linear staplers. The procedure's initiation involved using two 60-mm black cartridges with an Ethicon Echelon EndoFlex stapler. This was followed by using gold and blue cartridges, conducted over a 40 Fr calibration tube, extending straight to the angle of His. The staple line was reinforced by implementing seromuscular stitches, employing 3/0 PDS absorbable V-loc sutures at the upper part of the sleeve. Regarding the corpus and antrum, 2/0 non-absorbable PBT V-loc sutures were used (Covidien, Mansfield, MA, USA). The resected portion of the stomach was extracted from the peritoneal cavity via the left flank trocar site, with the possibility of muscle widening to facilitate specimen retrieval. No drainage was left in place. A slightly different technique was employed in non-IGB patients, as the gold and blue cartridges were used, and 3/0 absorbable PDS V-loc sutures reinforced the entire staple line. In some cases, concomitant repair of a hiatal hernia involved crural approximation with 2/0 non-absorbable PBT vloc sutures, and anterior stitches were applied at the 3, 9, and 12 o'clock positions, utilizing the same type of sutures.
