# Supplementary material for: Comparative Analysis of Laparoscopic Sleeve Gastrectomy with and Without Prior Endoscopic Intragastric Balloon Insertion: Examining Stomach Volumetry, Histopathologic Changes, Hormonal Levels, and Postoperative Outcomes
Source: Obes Surg. 2025 May 13;35(6):2039–52. doi: 10.1007/s11695-025-07907-4 (PMC12129852; doi:10.1007/s11695-025-07907-4)
Supplement: Supplementary file 2 — Supplementary file2 (DOCX 13 KB) [file 11695_2025_7907_MOESM2_ESM.docx]

**Appendix 2 multi-detector computed tomography (MDCT)**

Image acquisition was performed in the spine position and limited to the stomach, which is adequately inflated with gas on the topogram. Scans were acquired using the least radiation dose with the following parameters: 80 KV, 125 mA, 32 × 0.6 mm collimation, with 1 mm slice thickness reconstruction using SAFIRE iterative reconstruction. Data were transferred to a dedicated 3D workstation. Three-dimensional volume-rendering images were created by a combination of manual and semi-automatic segmentation tools. Different masks were created to represent the various relevant structures in different colors. The volume of the stomach (in preoperative series) and the sleeve pouch was measured on multiplanar reformations. Volume of the resected stomach was estimated by subtracting the pouch volume at 6 and 12 months postoperatively from the preoperative stomach volume, putting in consideration that intraoperative sleeve pouch construction was standardized throughout the study. The whole procedure was performed and interpreted in all patients by one radiologist
