# Supplementary material for: Comparative Analysis of Laparoscopic Sleeve Gastrectomy with and Without Prior Endoscopic Intragastric Balloon Insertion: Examining Stomach Volumetry, Histopathologic Changes, Hormonal Levels, and Postoperative Outcomes
Source: Obes Surg. 2025 May 13;35(6):2039–52. doi: 10.1007/s11695-025-07907-4 (PMC12129852; doi:10.1007/s11695-025-07907-4)
Supplement: Supplementary file 3 — Supplementary file3 (DOCX 14 KB) [file 11695_2025_7907_MOESM3_ESM.docx]

**Appendix 3: Hematoxylin and eosin Staining**

**Hematoxylin and eosin (H&E) Staining for Wall Thickness and Smooth Muscle Density Assessment**

Sleeve gastrectomy specimens were opened, rinsed with saline, and fixed in 10% formalin for at least 24 hours. Two perpendicular segments from the gastric body were processed into paraffin blocks, sectioned at 5 microns, and stained with hematoxylin and eosin (H&E). A blinded pathologist assessed the histology, with images captured at x40 magnification using a Leica EC4 digital camera attached to a microscope. ImageJ software measured mucosa and muscularis propria thickness. Ten high-power fields (HPFs) at x400 magnification were photographed to calculate smooth muscle cell density, excluding inflammatory and fibrous cells. Inflammation in the lamina propria was graded according to the Revised Sydney Classification (Figure 1A-B).

**Masson's Trichrome Staining for Gastric Wall Fibrosis**

Five-micron sections were stained with Masson's trichrome stain, highlighting fibrosis in blue and smooth muscle in red. Photographs were taken at x40 magnification, and ImageJ software quantified the blue-stained collagen as a percentage of the total area. At least five fields were analyzed, with the mean percentage reported (Figure 1C).

**Immunostaining for Ghrelin-Positive Cell Count**

Paraffin-embedded sections were stained with anti-Human Acylated Ghrelin antibody and counterstained with hematoxylin. After antigen retrieval, stained sections were examined at x200 magnification. Ghrelin-positive cells, identified by their brown granular cytoplasm, were counted using ImageJ across five fields per section. Results were expressed as the mean cell count per x200 field (Figure 1D).
