# Supplementary figures and images for: Comparative Analysis of Laparoscopic Sleeve Gastrectomy with and Without Prior Endoscopic Intragastric Balloon Insertion: Examining Stomach Volumetry, Histopathologic Changes, Hormonal Levels, and Postoperative Outcomes
Source: Obes Surg. 2025 May 13;35(6):2039–52. doi: 10.1007/s11695-025-07907-4 (PMC12129852; doi:10.1007/s11695-025-07907-4)

**Appendix 4: Flowchart**


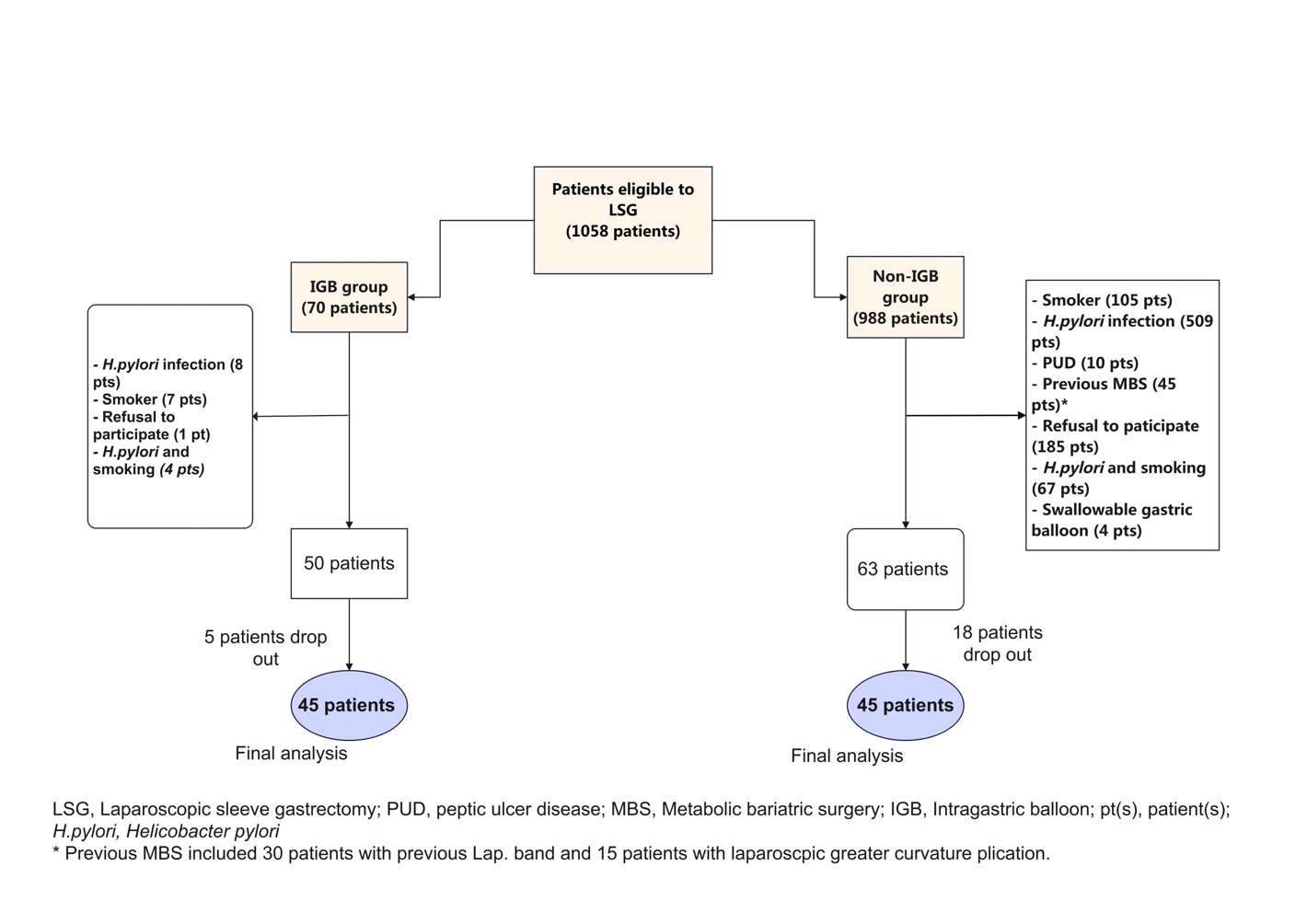

Supplement: Supplementary file 4 — Supplementary file4 (DOCX 182 KB) [file 11695_2025_7907_MOESM4_ESM.docx]
